# Hierarchical organization of social action features along the lateral visual pathway

**DOI:** 10.1016/j.cub.2024.01.064

**Published:** 2024-02-26

**Authors:** Emalie McMahon, Michael F. Bonner, Leyla Isik

After publication, we noted an error in the color bar label in Figures 2B and S2D–S2G stating that the brain maps reported the squared split-half reliability when the plotted data were not squared. We have corrected the color bar label in these plots. No other results were affected by the error, and the error does not affect the conclusions of the manuscript. We apologize for our mistake.

## Supplementary Material

1

## Figures and Tables

**Figure 2B.**
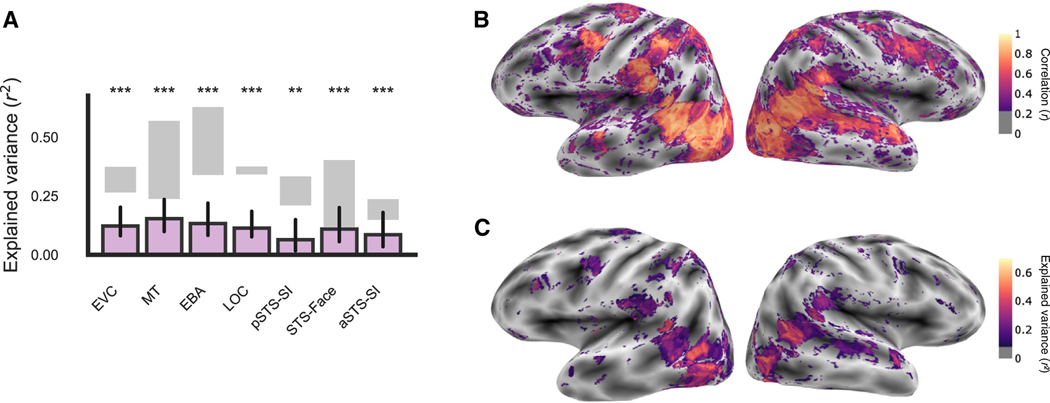
Extensive scanning yields high-quality fMRI data (corrected)

**Figure 2B.**
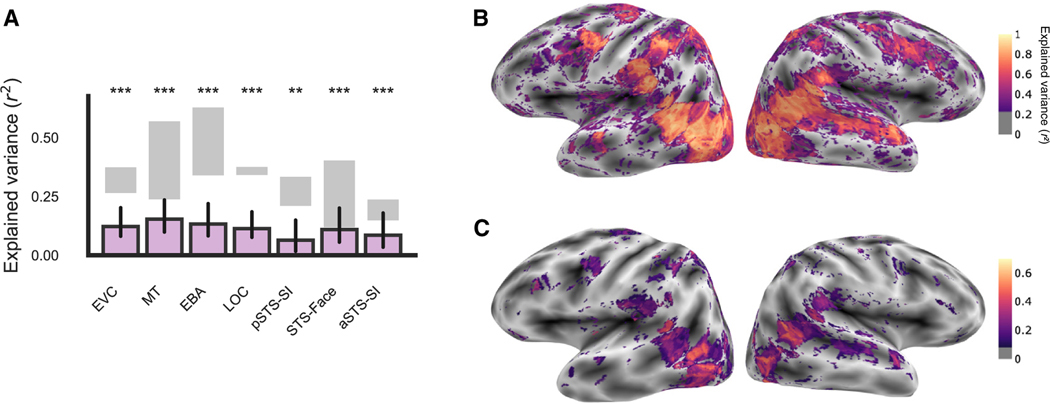
Extensive scanning yields high-quality fMRI data (original)

