# Supplementary material for: Hierarchical organization of social action features along the lateral visual pathway
Source: Curr Biol. Author manuscript; Available in PMC 2024 Apr 8. (PMC11000801; doi:10.1016/j.cub.2024.01.064)
Supplement: 1 [file NIHMS1968952-supplement-1.pdf]

Please cite this article in press as: McMahon et al., Hierarchical organization of social action features along the lateral visual pathway, Current Biology (2024), https://doi.org/10.1016/j.cub.2024.01.064

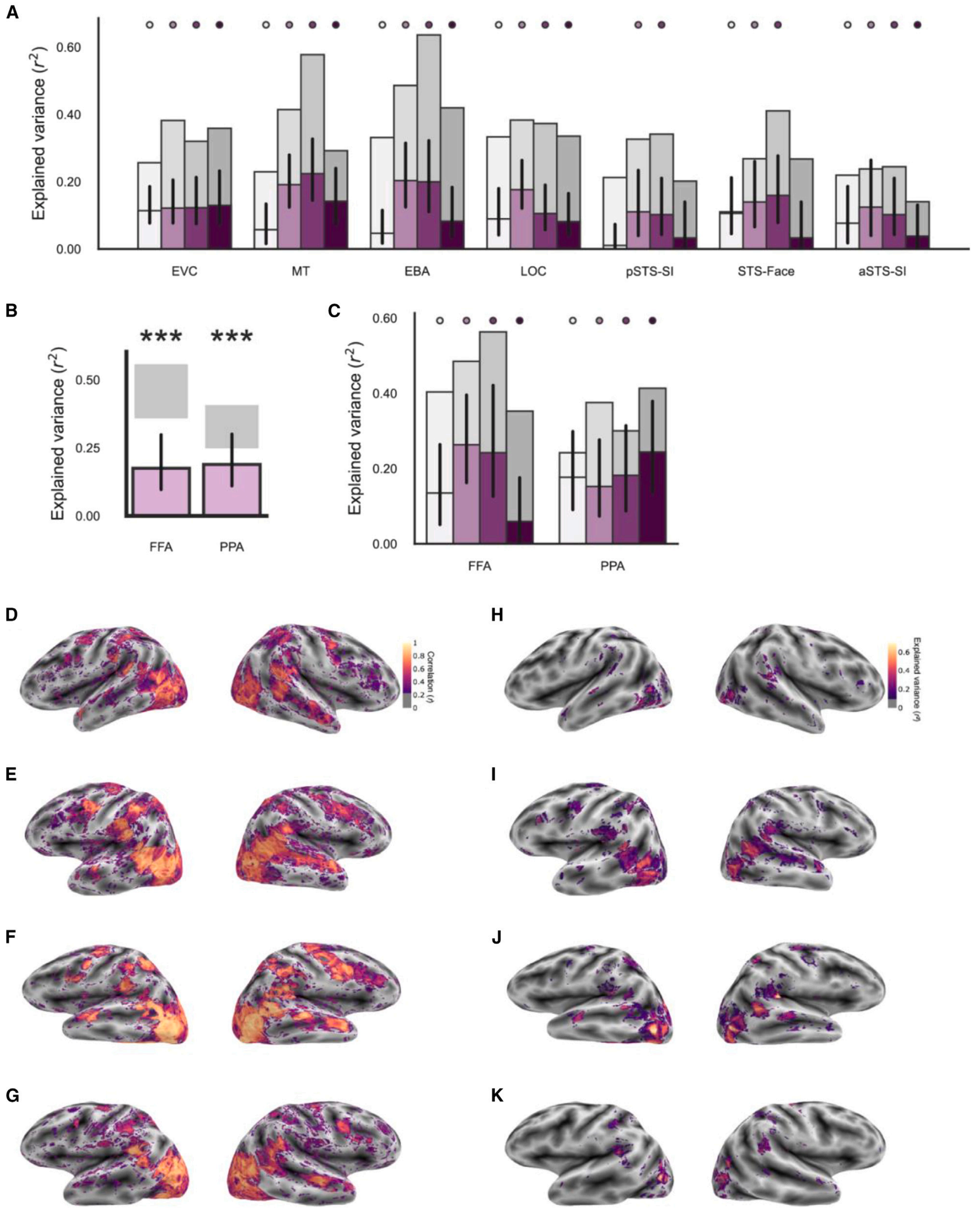

Figure S2. Reliability and encoding performance in individual subjects, Related to Figure 2. (corrected)

Please cite this article in press as: McMahon et al., Hierarchical organization of social action features along the lateral visual pathway, Current Biology (2024), https://doi.org/10.1016/j.cub.2024.01.064

CellPress
OPEN ACCESS

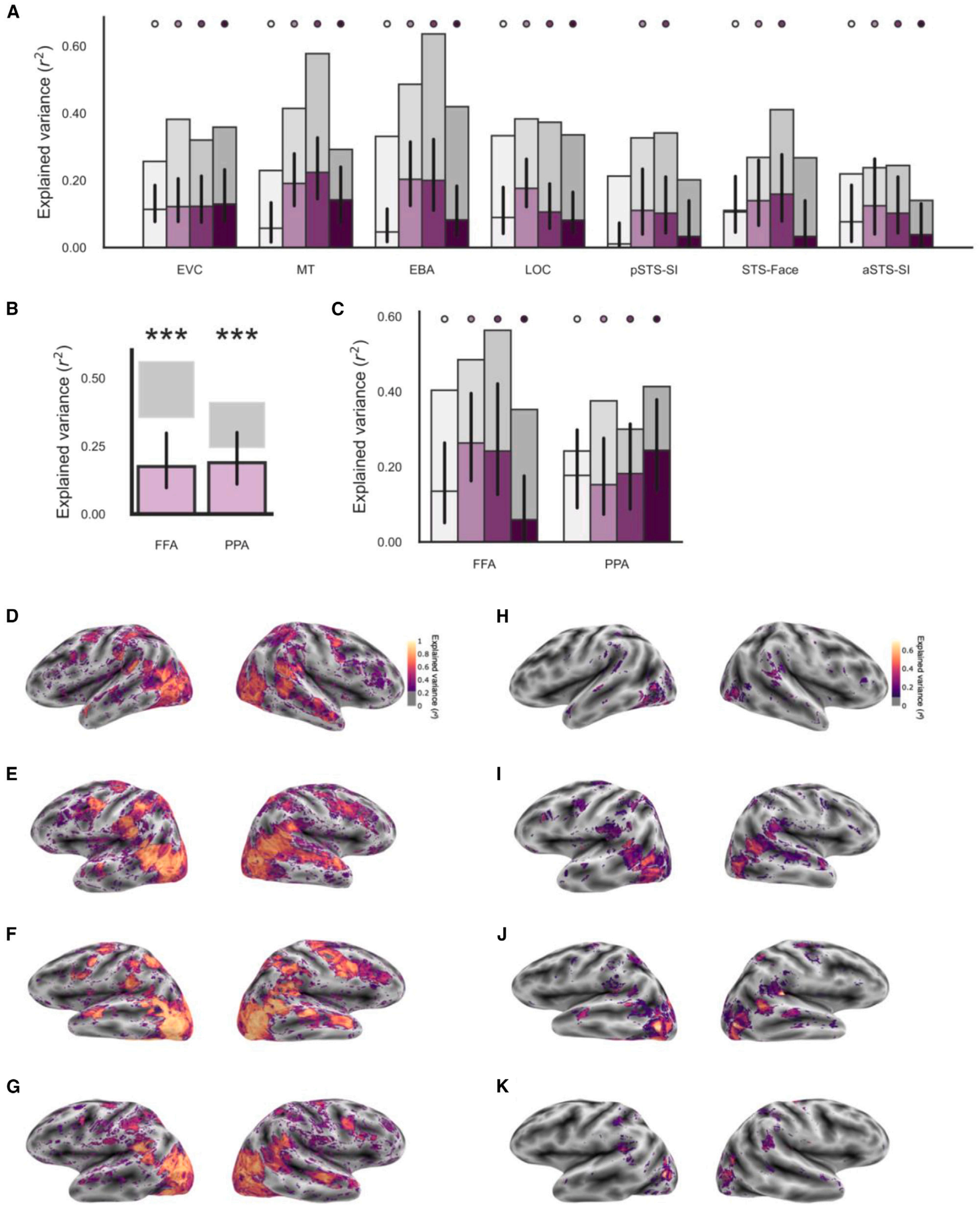

Figure S2. Reliability and encoding performance in individual subjects, Related to Figure 2. (original)
